# Poliprotect^®^, a Medical Device Made of Substances, Potently Protects the Human Esophageal Mucosa Challenged by Multiple Agents: Evidence from In Vitro and Ex Vivo Electrophysiological Models

**DOI:** 10.3390/ijms26020791

**Published:** 2025-01-18

**Authors:** Mohamad Khalil, Valeria Perniola, Elisa Lanza, Laura Mahdi, Pierluca Sallustio, Valeria Idone, Daniela Semeraro, Maria Mastrodonato, Mario Testini, Jean-Francois Desaphy, Piero Portincasa

**Affiliations:** 1 Clinica Medica “Augusto Murri”, Department of Precision and Regenerative Medicine and Ionian Area (DiMePrev-J), University of Bari Aldo Moro, 70124 Bari, Italy; mohamad.khalil@uniba.it (M.K.); valeria.perniola@uniba.it (V.P.); elisa.lanza1992@gmail.com (E.L.); laura.mahdi@uniba.it (L.M.); 2 Division of General Surgery, Department of Precision and Regenerative Medicine and Ionian Area (DiMePrev-J), University of Bari Aldo Moro, 70124 Bari, Italy; lucustio@hotmail.com (P.S.); mario.testini@uniba.it (M.T.); 3 Aboca S.p.A, Sansepolcro, Italy; vidone@aboca.it; 4 Department of Biosciences, Biotechnologies and Biopharmaceutics, University of Bari Aldo Moro, 70126 Bari, Italy; daniela.semeraro@uniba.it (D.S.); maria.mastrodonato@uniba.it (M.M.); 5 Section of Pharmacology, Department of Precision and Regenerative Medicine and Ionian Area (DiMePrev-J), University of Bari Aldo Moro, 70124 Bari, Italy; jeanfrancois.desaphy@uniba.it

**Keywords:** gastroesophageal reflux disease, esophageal mucosal integrity, synergistic molecular complex, electrophysiological protection, Ussing chambers, medical device made of substances

## Abstract

The integrity of esophageal epithelial cells in patients with gastroesophageal reflux disease (GERD) or GERD-like symptoms is the first mechanism of protection to decrease the sensitivity to gastric reflux and heartburn symptoms. We investigated the protective effects of Poliprotect^®^ (PPRO), a CE-marked medical device, on esophageal epithelial integrity using in vitro and ex vivo models. In vitro, the protective effects of PPRO were tested on Caco-2 cells. PPRO demonstrated safety and protection against oxidative damage induced by hydrogen peroxide. It also preserved epithelial integrity by maintaining transepithelial electrical resistance (TEER) against damage from calcium removal or bile acid exposure (taurodeoxycholic acid, TDCA). Ex vivo, esophageal biopsies from patients subjected to endoscopy were mounted in Ussing chambers and exposed to damaging agents (HCl or HCl + TDCA). Untreated biopsies (control) showed significant loss of epithelial resistance (up to −33%). In contrast, low concentrations of PPRO (50–100 µg/mL) provided strong protection against these damages (*p* < 0.001), even after 60 min of washing. Histological analysis confirmed the barrier-enhancing effect of PPRO. Overall, PPRO effectively protected the esophageal epithelium from damage in both models, suggesting its potential role in alleviating GERD or GERD-like symptoms by strengthening mucosal barriers and reducing epithelial sensitivity to reflux.

## 1. Introduction

Gastroesophageal reflux disease (GERD) is a condition characterized by the reflux of stomach contents into the esophagus. Although some degree of reflux is physiologic [1], intense and/or prolonged reflux can cause troublesome symptoms, i.e., heartburn, regurgitation, chest pain, water brash, globus sensation, odynophagia, and/or complications, i.e., Barrett’s esophagus, esophageal stricture, adenocarcinoma, chronic laryngitis, exacerbation of asthma [2]. GERD classification includes both nonerosive and erosive reflux disease, the latter indicating disease in the presence of endoscopically detectable damage of the esophageal mucosa.

GERD represents a burdensome clinical problem. It can occur at any age, including in children [3], and its prevalence ranges from 10 to 20% in Western countries, while it is less than 5% in Asia [4], with the most frequent symptoms being heartburn and regurgitation [5].

The management of GERD depends on the severity of the disease and includes lifestyle changes and pharmacotherapy as needed. Available medical treatments for GERD include proton pump inhibitors (PPIs), histamine receptor antagonists (H2-RAs), the novel potassium-competitive acid blockers (PCABs), prokinetics, and mucosal protectants, such as hyaluronic acid/chondroitin sulfate, alginates, and mucosal protective agents [6]. Treatment, however, must be tailored to specific cases, since many patients experience symptom relapse when acid suppression is stopped [7]. Additionally, the long-term use of gastric acid suppressants, particularly PPIs, is associated with an increased risk of certain complications such as gastric atrophy, impaired absorption of vitamin B and iron, enteric infections, acute interstitial nephritis, and osteoporosis [8]. While PPI use is associated with moderate changes to the upper and distal gut microbiota [9], there is no conclusive evidence to suggest that it leads to GI malignancies, myopathies, or acute coronary syndromes.

Mucosal protective agents play an evident role in alleviating chronic heartburn in patients with persistent or mild GERD symptoms despite acid suppressant therapy. Medical devices made of substances (MDMSs) such as mucosal protective agents for GERD treatment have attracted the attention of researchers and clinicians [10,11,12], but a rigorous evidence-based approach is still pending.

Interestingly, the Poliprotect^®^ formulation (PPRO) is a 100% natural MDMS acting as a protection agent in the esophagus and the stomach and helps counteract heartburn, pain, and acidic sensations. The mechanism likely includes the formation of a local barrier film on the mucosa that strengthens the mucosa’s inherent protective function by relieving and preventing irritation and inflammation caused by contact with gastric juices and other irritants. In a recent randomized and controlled clinical trial, PPRO proved non-inferior to standard-dose omeprazole in symptomatic patients with heartburn/ epigastric burning without erosive esophagitis and gastroduodenal lesions [13]. Being a 100% natural product, PPRO is a biodegradable and environmentally friendly product, an important aspect when dealing with the green economy approach. A large-scale and validated survey supports its safety and effectiveness in the treatment of common functional upper gastrointestinal disorders [14].

Experimental studies about the protective effects of PPRO against common mechanisms of damage are missing so far. Several studies have provided evidence that an impaired barrier function of the esophageal mucosa, as evidenced by epithelial dilated intercellular spaces, is a morphologic feature in both nonerosive and erosive GERD, suggesting that it is an early lesion in this disease and a fundamental defect in nonerosive reflux disease [15]. The integrity of epithelial surfaces is based on various cell–cell contacts that provide the structural basis for barrier function. Tight junctions, adherens junctions, and desmosomes are the three major structural units mediating barrier and sorting functions. Since the esophageal epithelium is constantly impacted by noxious agents, esophageal epithelial resistance plays a crucial role in protection. In line with this, we used a complementary translational approach to investigate the protective properties of PPRO in both cell culture models and human esophageal tissue by using viability assays and electrophysiological studies. Highly pathophysiologically relevant damaging factors were employed to induce epithelial barrier dysfunction and to test PPRO efficacy. The results point to a clear protective effect of the barrier created by PPRO against two of the main damage factors that cause GERD, i.e., gastric acid and bile acid.

## 2. Results

### 2.1. Protective Effect of uPPRO on Caco-2 Cell Viability Challenged with the Pro-Oxidant Agent H_2_O_2_

**Figure 1:**
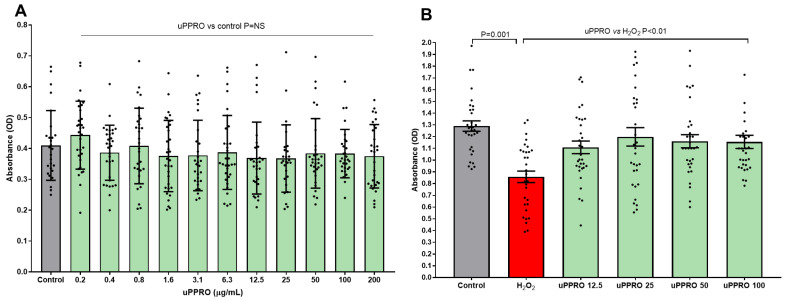
Cell viability of Caco-2 cells by MTT test under different conditions. (**A**) Increasing concentrations of uPPRO did not affect cell viability; (**B**) compared to control, H_2_O_2_ treatment decreased cell viability, which was restored by four increasing concentrations of uPPRO. Results are mean (horizontal line) and individual experiments (N = 32), with differences tested by ANOVA followed by Tukey multi-comparison test.

A pilot study investigated the effects of a wide range of concentrations of uPPRO, from 0.2 to 200 µg/mL, on Caco-2 cell viability. As shown in Figure 1A, uPPRO did not affect cell viability, compared to the control. Then, the protective effect of uPPRO was evaluated on H_2_O_2_-induced cellular oxidative damage. Caco-2 cells were treated with H_2_O_2_ (500 µM) alone or pre-treated with uPPRO for 3 h at different concentrations (12.5, 25, 50, and 100 µg/mL) (Figure 1B). After 24 h, cell viability was assessed. The untreated condition was also included. H_2_O_2_ alone induced a significant decrease (*p* = 0.001) in cell viability, corresponding to 38.5%, compared to the untreated control, whereas the uPPRO pre-treatments restored cell viability to the control values.

### 2.2. Preservation of the Permeability of a Caco-2 Cell Monolayer Treated with uPPRO and Challenged with TDCA: TEER Measurement

**Figure 2:**
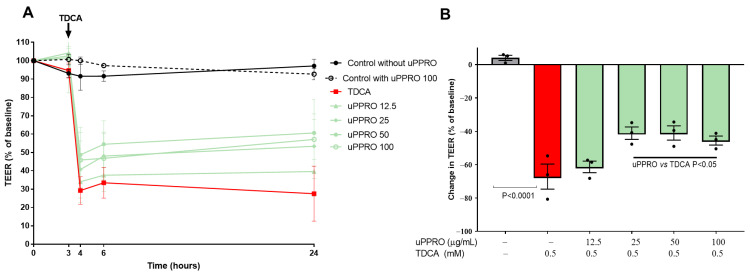
Transepithelial electrical resistance (TEER) in Caco-2 monolayers during 24 h. The experiments were performed in untreated cells (control) and in cells treated with uPPRO (12.5, 25, 50, and 100 µg/mL), TDCA (0.5 mM) alone, or a combination of uPPRO and TDCA. (**A**) TEER is expressed as a percent decrease with respect to the 100% baseline. (**B**) The graph focuses on the percent decrease in TEER at 24 h for each condition. uPPRO 25, 50, and 100 µg/mL were the most protective doses in response to TDCA damage. The values are mean values ± SEM from at least three independent experiments. Significant differences were tested by ANOVA followed by the Tukey multiple comparison test.

A Caco-2 cells monolayer underwent TEER measurement at baseline and during 24 h (Figure 2A). A pilot experiment confirmed the ability of the Caco-2 (untreated) monolayer to form a tight monolayer. The experiment also highlighted that the monolayer challenged with uPPRO 100 µg/mL maintained comparable TEER throughout 24 h. The damage control, represented by TDCA 0.5 mM, greatly reduced TEER (−71.2% of the basal value after 24 h, *p* < 0.0001), thus indicating the loss of monolayer integrity. On the contrary, the results highlighted the protective activity of uPPRO in preserving the integrity of the epithelium from a relevant damage condition at 25, 50, and 100 µg/mL (ranging from −45% to −50%) (Figure 2A,B).

### 2.3. Protective Effect of uPPRO on Caco-2 Barrier Function upon the Ca^2+^ and TDCA Challenge

**Figure 3:**
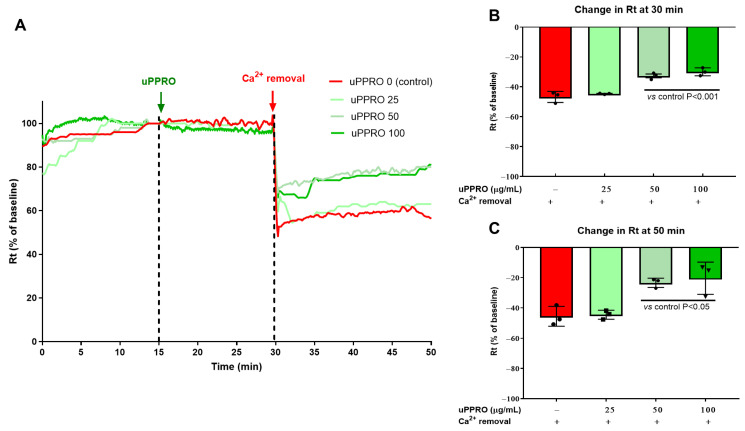
Effect of the apical addition of uPPRO on Caco-2 monolayers. (**A**) Representative traces (after 15 min of stabilization) of transepithelial resistance (Rt) after uPPRO addition at 25, 50, and 100 µg/mL for 15 min, followed by Ca^2+^ removal for 20 min. (**B**) Percentage changes in Rt induced by apical damage (Ca^2+^ removal) and (**C**) at the end of the experiment (20 min after damage). The results are expressed as percentages of the Rt basal values. The values are reported as mean values ± SEM. Statistical analysis was performed by one-way analysis of variance (ANOVA) with the Tukey multiple comparison test.

**Figure 4:**
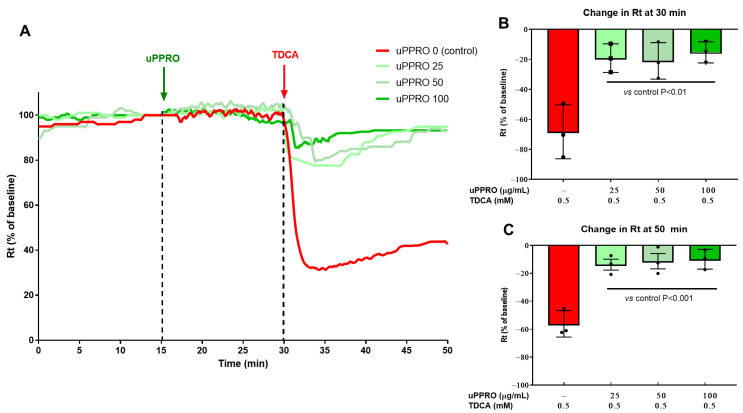
Effect of the apical addition of uPPRO on Caco-2 monolayers. (**A**) Representative traces (after 15 min of stabilization) of transepithelial resistance (Rt) after uPPRO addition at 25, 50, and 100 µg/mL, followed by the apical addition of TDCA (0.5 mM) for 20 min. (**B**) Percentage changes in Rt induced by apical damage (TDCA) and (**C**) at the end of the experiment (20 min after damage). The results are expressed as percentages of the Rt basal values. The values are reported as mean values ± SEM. Statistical analysis was performed by one-way analysis of variance (ANOVA) with the Tukey multiple comparison test.

The Ussing chamber was used to assess the electrophysiological properties of the Caco-2 barrier under two damaging conditions, i.e., Ca^2+^ removal from the buffer or TDCA addition to the buffer on the apical side (Figure 3 and Figure 4). Both experiments were performed in control (untreated) cells and in cells pre-treated with uPPRO following 15 min of stabilization. As shown in Figure 3A–C, transepithelial resistance (Rt) after stabilization (i.e., 15–30 min) was comparable in control cells and in cells pre-treated with uPPRO 25, 50, and 100 µg/mL. However, Ca^2+^ removal, after 30 min of stabilization, acutely decreased Rt compared to the baseline value (from 100% baseline value to −46.8%) On the contrary, the protective effect of uPPRO at 50 and 100 µg/mL (−32.6% and −29.9%, respectively, *p* < 0.01) persisted until the end of the experiment (50 min). Coherently with the TEER data, the damage control, represented by the solution of TDCA 0.5 mM, caused a dramatic reduction in Rt (−67.5%), as shown in Figure 4A–C. On the contrary, the results further confirmed the protective effect of the barrier created by the product, preserving the integrity of the Caco-2 cell monolayers (*p* < 0.05).

### 2.4. Effect of uPPRO and dPPRO on the Human Esophageal Barrier Following a HCl and TDCA Challenge in the Ussing Chamber

**Figure 5:**
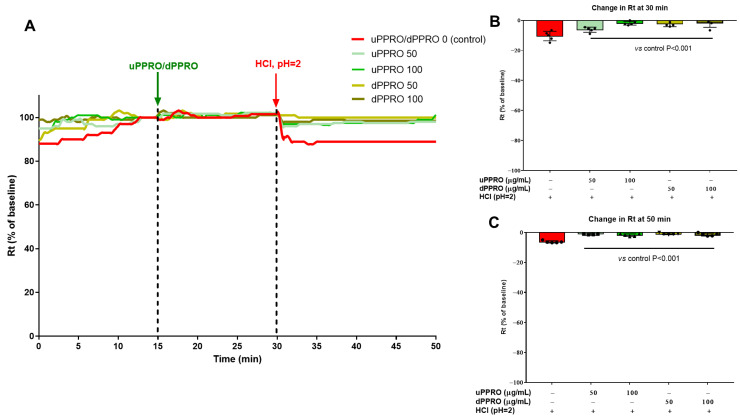
Effect of the apical addition of uPPRO and dPPRO on human esophageal biopsies. (**A**) Representative traces of transepithelial resistance (Rt) after uPPRO and dPPRO addition at 50 and 100 µg/mL for 15 min, followed by HCl damage (pH 2) for 20 min. (**B**) Percentage changes in Rt induced by the apical application of HCl damage at 30 min and (**C**) at the end of the experiment at 50 min. The results are expressed as percentages of the Rt basal values. The values are reported as mean values ± SEM of 5 tissues for each condition. Statistical analysis was performed by one-way analysis of variance (ANOVA) with the Tukey multiple comparison test.

**Figure 6:**
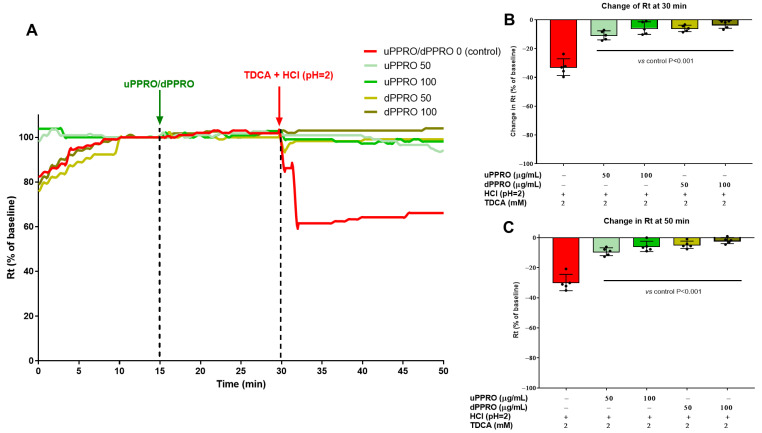
Effect of the apical addition of uPPRO and dPPRO on human esophageal biopsies. (**A**) Representative traces of transepithelial resistance (Rt) after uPPRO and dPPRO addition at 50 and 100 µg/mL for 15 min, followed by HCl + TDCA solution-induced damage (TDCA 2 mM, pH 2) for 20 min. (**B**) Percentage changes in Rt induced by the apical application of damage at 30 min and (**C**) at the end of the experiment at 50 min. The results are expressed as percentages of the Rt basal values. The values are reported as mean values ± SEM of 5 tissues for each condition. Statistical analysis was performed by one-way analysis of variance (ANOVA) with the Tukey multiple comparison test.

The protective effect of PPRO in terms of preservation of the integrity of the epithelial surface was further confirmed in ex vivo studies (Figure 5), using endoscopic biopsies. Two different solutions of PPRO, indicated as undigested (uPPRO) and digested (dPPRO), were used for these experiments. In the first experiment, resistance variation was evaluated after stabilization of esophageal tissues pre-treated with uPPRO or dPPRO at the concentrations of 50 and 100 µg/mL or left untreated (control condition). Notably, transepithelial resistance (Rt) remained comparable in both control and PPRO-treated tissues after stabilization (15–30 min). To induce damage to the human esophageal mucosa, the buffer solution was replaced with an acidic solution of HCl (pH = 2), and the resistance was again measured. At 30 min, the acid insult caused a 10.5% reduction in Rt compared to the untreated condition. On the contrary, the damage was completely prevented by the barrier created by PPRO, indicating efficient protection. Notably, the effect persisted until the end of the experiment (50 min). An equivalent experiment (Figure 6) was performed by treating the human esophageal tissue with a combination of HCl (pH = 2) plus TDCA (2 mM). Damage control showed a significant change in resistance, with a decrease in Rt of 33%. Pre-treatment with uPPRO and dPPRO prevented the damage until the end of the experiment, signifying, also in this challenge relevant conditions, that the barrier generated by the product could effectively protect from damage.

**Figure 7:**
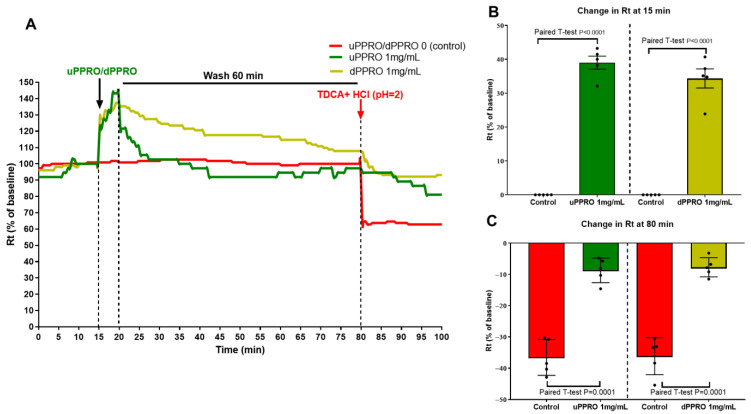
Effect of the apical addition of uPPRO and dPPRO on human esophageal biopsies from the same patients. (**A**) Representative traces of transepithelial resistance (Rt) after uPPRO and dPPRO addition at 1 mg/mL for 5 min, followed by a 60 min wash and then the apical addition of acidic TDCA (2 mM, pH 2) for 20 min. (**B**) Percentage changes in Rt induced by the apical addition of uPPRO and dPPRO at 15 min and (**C**) percentage change in Rt induced by the apical application of acidic TDCA damage at 80 min. The results are expressed as percentages of the Rt basal values. The values are reported as mean values ± SEM of 5 tissues for each condition. Statistical analysis was performed by the paired *T*-test.

An additional Ussing chamber experiment was performed, introducing a washing condition to evaluate the persistent protective effect of the layer created by the product (Figure 7A–C). Following 15 min of stabilization, the treatments with uPPRO and dPPRO at 1 mg/mL caused a significant (*p* < 0.05) increase in Rt (+39% and +34.4%, respectively) after 5 min compared to the control, highlighting, already at this stage, the protective activity of the product on human esophageal tissue. Despite the severity of the challenge (washing condition), the results showed that the barrier created by the product could effectively protect from damage caused by the addition of the HCl plus TDCA solution. In fact, the HCl plus TDCA solution at 80 min caused a 36.6% decrease in Rt, in contrast to the −8% decrease measured after treatment with uPPRO or dPPRO (*p* = 0.002). The effect of uPPRO was comparable to that of dPPRO.

The second experiment (Figure 6) showed that the combination of HCl (pH = 2) plus TDCA (2 mM) induced a decrease in Rt of 33%. Preincubation with uPPRO and dPPRO prevented this damage until the end of the experiment.

After this, the study aimed to investigate the behavior of the human esophagus in response to the addition of PPRO, the removal of PPRO, and a combined HCl plus TDCA treatment (Figure 7A–C). In this way, we simulated the persistent effects of PPRO after washing. Following 15 min of stabilization, the incubation with uPPRO and dPPRO at 1 mg/mL caused a significant (*p* < 0.05) increase in Rt (+39% and +34.4%, respectively) after 5 min compared to the control value. Upon washing, the effect of dPPRO on Rt persisted, compared to that of uPPRO. The addition of HCl + TDCA at 80 min to the untreated tissues caused a 36.6% decrease in Rt, which was greatly prevented by uPPRO or dPPRO (around −8% for both treatments, *p* = 0.002).

### 2.5. Histological Studies

**Figure 8:**
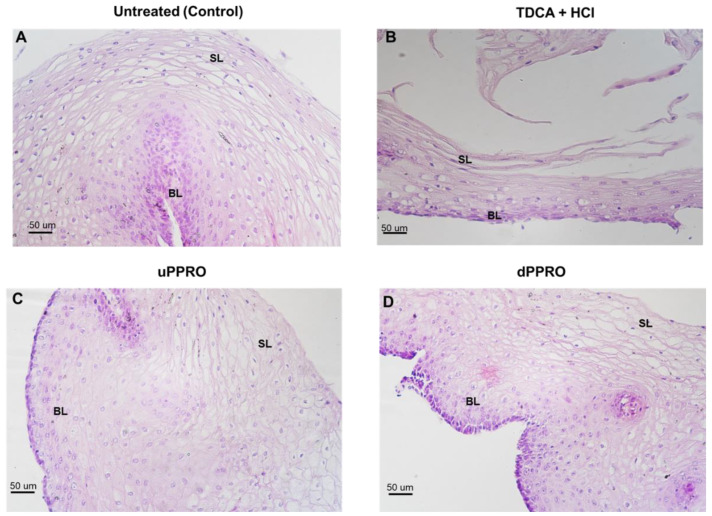
Microscopy analysis at 20x under a light microscope, showing the morphology of the tissues of esophageal biopsies stained with hematoxylin–eosin. (**A**) Control (untreated), (**B**) damage (TDCA+ HCl pH 2), (**C**) uPPRO treatment, and (**D**) dPPRO treatment at 100 µg/mL. Scale bar 50 um. BL, Basal layer; SL, superficial layer.

Following the Ussing chamber studies, histological analysis was conducted using light microscopy in untreated tissues (control) and in tissues treated with HCl + TDCA alone or pre-treated with uPPRO/dPPRO (Figure 8). We found that in the control tissue, the epithelial layer comprised a nonkeratinized stratified squamous epithelium, with its typical flat appearance and with flatter cells as they moved away from the base toward the apical portion, showing a healthy phenotype. After HCl + TDCA treatment, the esophageal tissue showed mucosal damage, including loss of epithelial cells, swelling, widening of the spaces between the layers, and disruption of the normal architecture. Pre-incubation with uPPRO or dPPRO rescued the tissue from epithelial damage by restoring an epithelial layer somewhat similar to that of the control (undamaged) esophagus, with widespread hyperplasia of the superficial cells.

## 3. Discussion

In this study, we used several in vitro and ex vivo models and provide evidence that Poliprotect (PPRO), a 100% natural synergetic molecular complex, prevents different types of damage such as those caused by acid and detergent bile acid on epithelia, protecting esophageal integrity. PPRO is designed as a medical device composed of a synergistic combination of polysaccharides, minerals, and flavonoids, specifically formulated to protect esophageal integrity against refluxed materials. The observed protective effects in clinical [13] and this current experimental studies are attributed to the complex as a whole, rather than to its individual components. This highlights the importance of the interaction between the ingredients in achieving the desired therapeutic outcomes.

Treating upper GI disorders including GERD symptoms can be quite challenging. The treatment typically relies on PPIs, prokinetics, and neuromodulators, along with herbal substances and even alternative medicine [16]. The prolonged use of PPIs is associated with an increased risk of side effects and potential interactions with concomitant therapies. In addition, young adults, children, and pregnant women with mild symptoms may be over-treated with these drugs, with risks outweighing the benefits. Mucosal protective agents (MPAs) are also commonly used, either alone or in combination with PPIs, and have been effective in reducing symptoms [17]. The results of this study highlight that Poliprotect is able to create a protective barrier that protects the esophageal mucosa. The 3D epithelium cell model closely mimicked the normal barrier integrity and function. In epithelial cells, we found that Poliprotect showed cytoprotective effects and protected cell monolayers against a relevant damage condition represented by a pro-oxidant insult. [18,19,20,21]. Moreover, we found that PPRO protected against bile acid damage mimicking the damage caused by the duodenum–gastro–esophageal reflux of toxic bile acids secreted across the hepato–biliary tract [22]. Although the cell culture model is more susceptible to damage compared to native biopsy tissue, we used a less aggressive test solution (TDCA 0.5 mM, vs. 2 mM for tissues). This increased vulnerability allowed the model to serve as a sensitive indicator of mucosal protection. Indeed, Poliprotect prevented the damage caused by bile acid likely by physical interactions. In addition, changes in the disruptionof tight-junction proteins due to Ca^2+^ removal were evident (>40% loss of barrier resistance). The integrity of the epithelium was completely protected by the film created by the product. However, the accumulative and synergetic effects of PPRO on cellular and ex vivo models of epithelial integrity were not tested before. In this study, we ensured that the viscosity of the Poliprotect solution was carefully managed to prevent it from being a confounding factor, as high viscosity could create a physical barrier independently of the test solutions. Indeed, Poliprotect was well dissolved in water without any increase in the solution viscosity. Additionally, a thorough 60 min washing of the product pre-treatment solutions was performed to ensure accurate results and efficacy and to confirm that Poliprotect may confer benefits via its physical properties. Notably, the exposure of esophageal biopsies to Poliprotect markedly increased transepithelial resistance. This finding provided further evidence of Poliprotect physical adherence to the luminal surface. This was confirmed by the capability of the product to protect the esophageal tissue also when subjected to a washing condition. After 60 min of washing, the PPRO pre-treatment prevented the reductions in the esophageal biopsies Rt caused by exposure to acidic solutions with bile acid; this demonstrated the adhesive potential of PPRO, a property determined primarily by its natural polysaccharides. These results suggest that a strategically timed topical application of PPRO, such as immediately after meals, may help reduce mucosal injury by reflux. In humans, factors like gravity, saliva, and mucus can dislodge PPRO from the esophageal lining. To counteract this, we employed orally digested PPRO and applied vigorous washing to ensure that PPRO did not adhere to the mucosa because of stasis and that pre-digested PPRO would be able to exhibit the same efficacy on the human esophagus. PPRO appeared to become adhesive after hydration and oral digestion. When adhered to the esophageal epithelium, PPRO enabled protection against refluxate damage. The adhesive properties together with the functional proprieties appeared to ensure PPRO effectiveness even at lower concentrations of PPRO (i.e., 50 and 100 ug/mL), and this confirmed that PPRO ingestion may also be useful in long-term protection, where PPRO can exert preventive and cyto- and mucosa-protective effects besides its antioxidant and adhesive effects.

This study has some limitations. The in vitro model was used as a physiological approach to inspecting the adhesive proprieties of PPRO and its possible effect on epithelial cells and adherence (tight-junction proteins) and not as a true reflection of the esophagus physiological conditions. The application of acidic damage, which can reflect the in vivo damage occurring in GERD, was not fully implementable, since an acidic solution with pH 2 was associated with mild damage (i.e., −10% of basal Rt). Nevertheless, protection by PPRO was still evident. Thus, the PPRO effect on esophageal integrity does not necessarily translate to a therapeutic effect in vivo but certainly integrates the previous data on the clinical potential of PPRO in reducing GERD or GERD-like symptoms. In addition, these data suggest the use of PPRO as a co-treatment approach for GERD based on topical mucosal protection. Despite PPRO currently having limited global availability, this study aimed to present novel insights into its protective mechanisms against esophageal damage using well-established electrophysiological methods. The findings may stimulate further research and discussions about its therapeutic potential, as well as encourage efforts to improve its accessibility in the future. Additionally, the methods we used in this study can serve as a foundation for evaluating new products to determine their adherence and protective properties before applying them for a clinical evaluation. The determination of clinically relevant concentrations in experimental studies poses inherent challenges, particularly when translating in vitro findings to in vivo conditions. In this study, we aimed to simulate the clinical context by approximating the physiological concentration of PPRO based on its dosage, the estimated saliva volume during swallowing, and PPRO bioavailability, arriving at 1 mg/mL as the primary test concentration. While this calculation provides a reasonable representation of PPRO clinical exposure, it is important to acknowledge that the experimental conditions, including the washing protocols and static test environments, did not fully replicate the dynamic physiological processes in vivo. Furthermore, to gain a broader understanding of the mechanistic effects, we tested additional concentrations that may not directly correspond to clinical scenarios. These findings provide valuable insights but also highlight the need for further dose translation studies in more physiologically relevant systems, such as in vivo models, to bridge the gap between experimental outcomes and clinical applications.

In conclusion, PPRO topical application acted on the human esophageal mucosa by contrasting different and pathophysiologically relevant types of damage such as that caused by acid/bile acid for an extended period in an ex vivo model. The effect appeared to occur via adhesion on the mucosal surface and the antioxidant and cytoprotective effects of the Poliprotect^®^ formulation. These results further strengthen the clinical results on the efficacy and safety of the Poliprotect formulation for the treatment of GERD symptoms.

## 4. Materials and Methods

**Figure 9:**
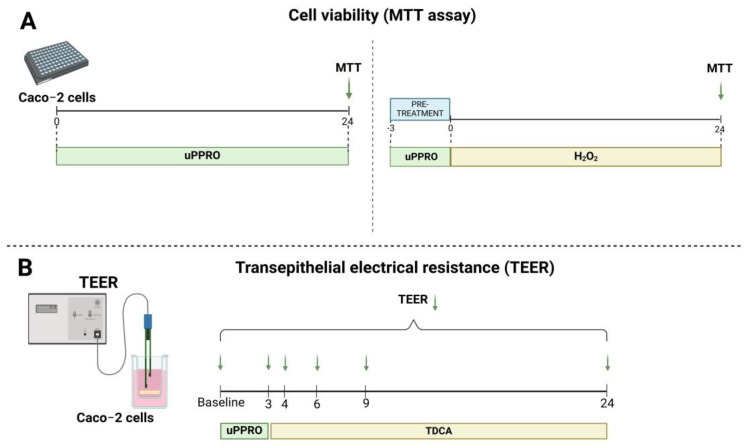
Study design; (**A**) MTT cell viability assay, (**B**) transepithelial electrical resistance (TEER) method.

**Figure 10:**
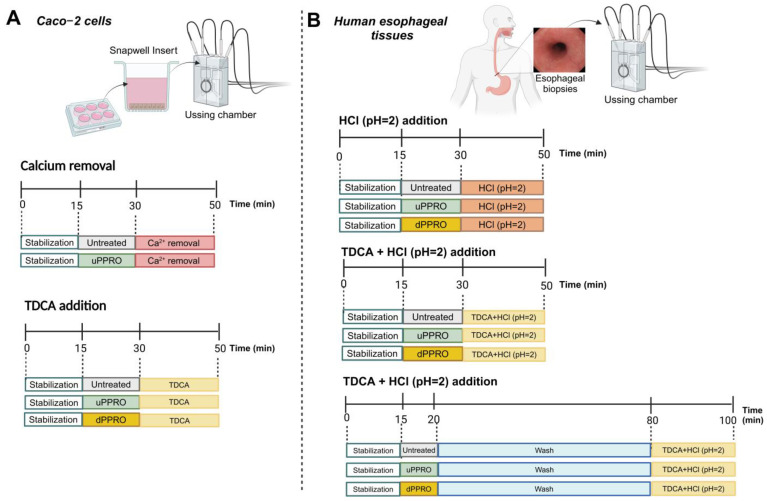
Electrophysiological studies; (**A**) Ussing chamber experiments with Caco-2 monolayers, (**B**) Ussing chamber experiments with human esophageal biopsies.

The in vitro Caco-2 cell model is an excellent biophysical system to mimic the human epithelial barrier. The translational value of the experimental protocol was tested by the ex vivo tissue model of human esophageal biopsies. Both cells and tissues were exposed to agents mimicking gastroesophageal reflux and damage (i.e., HCl and bile acid, alone and in combination) and to PPRO, mimicking protection. The experimental design is depicted in Figure 9 and Figure 10.

### 4.1. PPRO Preparation

PPRO, a CE-marked medical device (NeoBioanacid, Aboca S.p.A, Sansepolcro, Italy), is a 100% natural medical device made of Poliprotect^®^ (a polysaccharide fraction from *Aloe vera*, *Malva sylvestris*, and *Althea officinalis*; minerals, limestone and nahcolite) and a flavonoid fraction from *Glycyrrhiza glabra* and *Matricaria recutita*. PPRO was prepared from a 1.5 g tablet to obtain a final concentration of the functional fraction of polysaccharide of 1.25 mg/mL. Whenever relevant, two different solutions of PPRO were tested:

- Undigested PPRO (uPPRO): One PPRO tablet was dissolved in 30 mL of double-distilled water (ddH_2_O). The solution was shaken for 10 min until PPRO was thoroughly dissolved and centrifuged at 14,000 rpm for 10 min. The supernatant was transferred to a sterile Eppendorf tube and stored at −20 °C for future use.
- Digested PPRO (dPPRO): One PPRO tablet was first pre-treated with 1 mL of human saliva from 3 healthy donors who had not taken drugs or probiotics for at least the last three months, then incubated for 5 min at 37 °C, and then dissolved in 29 mL of ddH_2_O. Then, the procedure was similar to that for uPPRO preparation.

To approximate the minimal concentration of PPRO that reflects its clinical use, we based our calculations on the composition of the product and on physiological parameters. PPRO is available in tablets with 40 mg of polysaccharide functional parts. According to the manufacturer, 1 tablet is dissolved in saliva in the mouth in about 10 min, meaning a final volume of saliva of about 5–10 mL. Taking into account the bioavailability of PPRO, we conservatively used a concentration of PPRO as low as 1 mg/mL to evaluate the adherence of PPRO on the esophageal mucosa after one hour of washing. In cellular models, we confirmed that concentrations up to 0.2 mg/mL were not cytotoxic. This was the reason to use a range of concentrations of PPRO from 0 to 0.2 mg/mL. This multi-tiered approach allowed us to explore both the functional properties of PPRO and its dose–response relationship in the experimental setting ranging from cellular to mucosal models.

### 4.2. In Vitro Model

#### 4.2.1. Cell Culture

For the in vitro studies, the human colon adenocarcinoma Caco-2 (HTB-37) cell line was kindly provided by Prof. Rosa Caroppo, University of Bari, Italy. The cells were grown and maintained in DMEM GlutaMAX supplemented with 10% fetal bovine serum,1% penicillin/streptomycin solution, and 1% non-essential amino acids, at 37 °C in a humidified atmosphere containing 5% CO_2_. All reagents were purchased from Thermo Fisher Scientific Inc. (Waltham, MA, USA).

#### 4.2.2. Cell Viability

Caco-2 cell viability was assessed by the 3-(4,5-dimethylthiazol-2-yl)-2,5 diphenyl-tetrazolium bromide (MTT) assay [23]. Briefly, the cells were seeded in a 96-well plate (10^4^ cells per well), in complete medium. After reaching 70–80% of confluency, to exclude any possible cytotoxic effects, the cells were treated with increasing concentrations (0–200 µg/mL) of uPPRO for 24 h. For the cytoprotection effect of uPPRO, the cells were first incubated for 3 h with uPPRO (0, 12.5, 25, 50, and 100 µg/mL) and then insulted with the pro-oxidant agent hydrogen peroxide at 500 µM for 24 h. At the indicated times, 20 µL of MTT reagent (5.0 mg/mL) was added to each well, followed by incubation in the dark at 37 °C for 4 h. Then, the unreacted MTT dye was removed by aspiration, and 100 µL of acidified isopropanol was added to solubilize the purple formazan crystals within metabolically active cells. The absorbance was recorded at 570 nm by an optical plate reader (BioTek 800 TS Absorbance Reader; Agilent, Santa Clara, CA, USA).

#### 4.2.3. Transepithelial Electrical Resistance (TEER)

For TEER measurement, Caco-2 cells (2.2 × 10^5^ cells/cm^2^) were seeded on Transwell^®^ (Fisher Scientific, Segrate, Italy) inserts, changing the medium every 3 days. The cells were differentiated for 21 days and continually monitored via inverted microscopy (ZEISS Axiovert 100, Oberkochen, Germany) [24]. After 21 days, cell monolayers with an initial TEER of 800–1000 Ω cm^2^ were used to ensure intact Caco-2 monolayers with well-developed tight junctions. To inspect the possible protective effects of uPPRO, Caco-2 monolayers were pre-treated with uPPRO for 3 h and then incubated with the detergent agent taurodeoxycholic acid (TDCA) at 0.5 mM. TEER was recorded at baseline, after 3 h of uPPRO incubation, and after damage at 1, 3, 6, and 21 h using an EVOM2 Epithelial Tissue Volt/Ohmmeter (Scientific Instruments, Simmerath, Germany).

#### 4.2.4. Ussing Chamber

The electrophysiological studies were performed in the Ussing chamber (Scientific Instruments, Simmerath, Germany), a system to measure the transport of ions across cells. Caco-2 cells were seeded at a density of 2.2 × 10^5^ cells/cm^2^ on Snapwell™ supports (Fisher Scientific, Segrate, Italy) with 1.12 cm^2^ of cell growth area. Caco-2 cells were grown as monolayers for 21 days. Therefore, the inserts were mounted into the Ussing chamber. The monolayers were mounted in the chambers provided with apical and basolateral sides, bathed with a volume of 3 mL of Krebs–Ringer solution containing, in mM concentrations, 115 NaCl, 25 NaHCO_3_, 0.4 KH_2_PO_4_, 2.4 K_2_HPO_4_, 1.2 MgCl_2_, 1.2 CaCl_2_, 10 glucose (pH 7.4, 300 mOsm) on each side, and continuously oxygenated with O_2_/CO_2_ (95%/5%), circulated by gas lift. Two pairs of Ag/AgCl electrodes were used to monitor either the transmural potential difference (PD, mV) under open-circuit conditions or the short-circuit current (I_SC_, µA/cm^2^) with the transmural PD clamped to zero. Changes in I_SC_ depend on the net active ion transport across the epithelium. Transepithelial resistance (Rt, Ω·cm^2^) was calculated according to Ohm’s law to provide an overall measurement of cell monolayer integrity. A decreasing Rt value indicates increased permeability. Simultaneous experiments were conducted in four PC-controlled chambers (software Clamp v. 2.14, Aachen, Germany). We employed the Ca^2+^ switch assay to study epithelial tight junction and barrier function in Ussing chamber-mounted epithelial monolayers [25]. After stabilization in the Ringer buffer (~15 min), the monolayers were treated apically with uPPRO (0, 25, 50, and 100 µg/mL) for 15 min. Afterward, the buffer solution was replaced on both sides (apical and basolateral) with Ca^2+^-free Ringer buffer containing EDTA. After this exposure, Rt was continuously monitored for 20 min. Furthermore, we employed TDCA as a damaging agent. Caco-2 monolayers were treated with uPPRO (0, 25, 50, and 100 ug/mL) for 15 min, followed by damage (TDCA 0.5 mM) for 20 min. Transepithelial resistance (Rt) was measured with a voltage clamp protocol, as previously described [26].

### 4.3. Ex Vivo Model

#### 4.3.1. Human Esophageal Tissues

For the ex vivo studies, esophageal tissues were collected from the esophageal biopsies of 47 patients (M/F 26/17, mean BMI 27.1, range 22.8–33.9 kg/m^2^; mean age 58.0, range 31–87 years) scheduled for routine esophago–gastroduodenoscopy (EGDS) for symptoms typical of gastroesophageal reflux, i.e., heartburn, regurgitation. Patients with known gastrointestinal inflammation or tumors and gastric/duodenal ulcers were excluded from the study. Overall, 35 patients with a normal esophageal mucosa were included. Nine patients had macroscopically evident esophagitis confirmed at histology, while for three other patients, the samples were inadequate for the study. EGDS was performed under midazolam and hyoscine medication and previous lidocaine spray application through the mouth. After the exploration of the entire esophagus, two esophageal mucosal biopsies were taken using a biopsy punch of 2.3 mm from 1 cm above the squamocolumnar junction (Z-line). The tissue was immediately placed in a preoxygenated and iced Krebs–Ringer buffer solution at pH 7.4. The biopsies were rapidly transported to the laboratory for the electrophysiological studies. All biopsies for the following studies were taken by the same endoscopist (LS) using the same technique. The study obtained ethical approval by the local ethical committee (Study number 7689, Protocol number 0030626/28/03/2023). All participants signed a written informed consent form.

#### 4.3.2. Ussing Chamber

The experiments investigated the protective effects of uPPRO and dPPRO on HCl- and HCl–bile acid-induced damage in tissues mounted in Ussing chambers according to an open-circuit protocol [27]. Two biopsies from each patient were used to study damage and protection. Biopsies were orientated under a microscope (Nikon SMZ745T, Japan) and mounted in Ussing chambers with an opening surface area of 0.017 cm² and then stabilized to reach a stable basal Rt (around 15 min).

A first experiment was designed to investigate the short-term protective effects of low concentrations of uPPRO and dPPRO on electrophysiological parameters. The biopsies were incubated with 50 and 100 µg/mL of uPPRO and dPPRO, diluted in Ringer solution, pH 7.4. After 15 min, damage was induced by adding HCl in Ringer solution to reach pH = 2 or TDCA (2 mM) + HCl to reach pH = 2. Both conditions can mimic esophageal reflux. A second experiment was designed to investigate the long-term protective effects of high concentrations of uPPRO and dPPRO on electrophysiological parameters. For this, the Ringer solution was removed, and uPPRO/dPPRO solutions were added at a high concentration (1 mg/mL, diluted in Ringer solution, pH 7.4) for 5 min on the apical side. This protocol mimicked the condition of both undigested and digested PPRO reaching the esophageal epithelium. Afterwards, the PPRO-containing solution was washed off with pre-warmed and oxygenated Ringer buffer (pH 7.4), and the samples were maintained a further 60 min before being challenged with TDCA 2 mM + HCl (pH 2) for another 20 min. Rt was continuously measured. For both experiments, the percentage change in Rt from the baseline (Rt value after 15 min stabilization) was calculated after damage and at the end of the experiment (20 min after damage).

### 4.4. Histological Studies

After the Ussing chamber studies, in order to check for morphological damage, esophageal tissues samples of both untreated and treated samples were fixed in 10% neutral buffered formalin, washed in 0.1 M saline phosphate buffer (PBS) at pH 7.4, dehydrated in a graded series of ethanol, diaphanized in Histolemon (Carlo Erba Reagents, Cornaredo, Milan, Italy), and embedded in paraffin wax. Serial sections, 5 µm thick, were cut using a rotary microtome (Leica RM 2155, Leica, Wetzlar, Germany). Then, sections were stained with hematoxylin–eosin to assess the morphology of the tissues. Images were captured using a Nikon Eclipse E600 light microscope equipped with a DS-Fi3 microscope camera (Nikon Instruments Ltd., Campi Bisenzio, Florence, Italy).

### 4.5. Statistical Analysis

All data represent means ± standard error (SEM). Comparisons between two groups were made with paired or unpaired *t*-test where appropriate. Multiple comparisons were made with analysis of variance (one-way ANOVA) followed by Tukey multiple comparison test. Significance was declared at *p* < 0.05. Statistical analyses and graph visualization were performed using GraphPad Prism version 8.0 (GraphPad Software, San Diego, CA, USA).

## Data Availability

The raw data supporting the conclusions of this article will be made available by the authors on request.

## References

[1] Richter J.E. (1996). Typical and atypical presentations of gastroesophageal reflux disease. The role of esophageal testing in diagnosis and management. Gastroenterol. Clin. N. Am..

[2] Vakil N., van Zanten S.V., Kahrilas P., Dent J., Jones R. (2006). The Montreal definition and classification of gastroesophageal reflux disease: A global evidence-based consensus. Am. J. Gastroenterol..

[3] Tighe M., Afzal N.A., Bevan A., Hayen A., Munro A., Beattie R.M. (2014). Pharmacological treatment of children with gastro-oesophageal reflux. Cochrane Database Syst. Rev..

[4] Dent J., El-Serag H.B., Wallander M.A., Johansson S. (2005). Epidemiology of gastro-oesophageal reflux disease: A systematic review. Gut.

[5] Camilleri M., Dubois D., Coulie B., Jones M., Kahrilas P.J., Rentz A.M., Sonnenberg A., Stanghellini V., Stewart W.F., Tack J. (2005). Prevalence and socioeconomic impact of upper gastrointestinal disorders in the United States: Results of the US Upper Gastrointestinal Study. Clin. Gastroenterol. Hepatol..

[6] Visaggi P., Bertin L., Pasta A., Calabrese F., Ghisa M., Marabotto E., Ribolsi M., Savarino V., de Bortoli N., Savarino E.V. (2024). Pharmacological management of gastro-esophageal reflux disease: State of the art in 2024. Expert Opin. Pharmacother..

[7] Fass R., Sifrim D. (2009). Management of heartburn not responding to proton pump inhibitors. Gut.

[8] Yibirin M., De Oliveira D., Valera R., Plitt A.E., Lutgen S. (2021). Adverse Effects Associated with Proton Pump Inhibitor Use. Cureus.

[9] Macke L., Schulz C., Koletzko L., Malfertheiner P. (2020). Systematic review: The effects of proton pump inhibitors on the microbiome of the digestive tract-evidence from next-generation sequencing studies. Aliment. Pharmacol. Ther..

[10] Savarino V., Marabotto E., Zentilin P., Furnari M., Bodini G., Giovanni Giannini E., Vincenzo Savarino E. (2023). Gastrointestinal functional disorders can benefit from the use of medical devices made of substances. Front. Drug Saf. Regul..

[11] Salehi M., Karegar-Borzi H., Karimi M., Rahimi R. (2017). Medicinal Plants for Management of Gastroesophageal Reflux Disease: A Review of Animal and Human Studies. J. Altern. Complement. Med..

[12] Hosseinkhani A., Lankarani K.B., Mohagheghzadeh A., Long C., Pasalar M. (2018). An Evidence-based Review of Medicinal Herbs for the Treatment of Gastroesophageal Reflux Disease (GERD). Curr. Drug Discov. Technol..

[13] Corazziari E.S., Gasbarrini A., D’Alba L., D’Ovidio V., Riggio O., Passaretti S., Annibale B., Cicala M., Repici A., Bassotti G. (2023). Poliprotect vs Omeprazole in the Relief of Heartburn, Epigastric Pain, and Burning in Patients Without Erosive Esophagitis and Gastroduodenal Lesions: A Randomized, Controlled Trial. Am. J. Gastroenterol..

[14] Cioeta R., Muti P., Rigoni M., Morlando L., Siragusa F., Cossu A., Giovagnoni E. (2022). Effectiveness and tolerability of Poliprotect, a natural mucosal protective agent for gastroesophageal reflux disease and dyspepsia: Surveys from patients, physicians, and pharmacists. Front. Drug Saf. Regul..

[15] Barlow W.J., Orlando R.C. (2005). The pathogenesis of heartburn in nonerosive reflux disease: A unifying hypothesis. Gastroenterology.

[16] Harer K.N., Hasler W.L. (2020). Functional dyspepsia: A review of the symptoms, evaluation, and treatment options. Gastroenterol. Hepatol..

[17] Scally B., Emberson J.R., Spata E., Reith C., Davies K., Halls H., Holland L., Wilson K., Bhala N., Hawkey C. (2018). Effects of gastroprotectant drugs for the prevention and treatment of peptic ulcer disease and its complications: A meta-analysis of randomised trials. Lancet Gastroenterol. Hepatol..

[18] Murugan S.K., Bethapudi B., Raghunandhakumar S., Purusothaman D., Nithyanantham M., Mundkinajeddu D., Talkad M.S. (2022). A flavonoid rich standardized extract of Glycyrrhiza glabra protects intestinal epithelial barrier function and regulates the tight-junction proteins expression. BMC Complement. Med. Ther..

[19] Sebai H., Jabri M.A., Souli A., Hosni K., Rtibi K., Tebourbi O., El-Benna J., Sakly M. (2015). Chemical composition, antioxidant properties and hepatoprotective effects of chamomile (*Matricaria recutita* L.) decoction extract against alcohol-induced oxidative stress in rat. Gen. Physiol. Biophys..

[20] Hwang S.H., Wang Z., Guillen Quispe Y.N., Lim S.S., Yu J.M. (2018). Evaluation of Aldose Reductase, Protein Glycation, and Antioxidant Inhibitory Activities of Bioactive Flavonoids in *Matricaria recutita* L. and Their Structure-Activity Relationship. J. Diabetes Res..

[21] Jabri M.A., Aissani N., Tounsi H., Sakly M., Marzouki L., Sebai H. (2017). Protective effect of chamomile (*Matricaria recutita* L.) decoction extract against alcohol-induced injury in rat gastric mucosa. Pathophysiology.

[22] Portincasa P., Moschetta A., Palasciano G. (2006). Cholesterol gallstone disease. Lancet.

[23] Khalil M., Khalifeh H., Baldini F., Serale N., Parodi A., Voci A., Vergani L., Daher A. (2020). Antitumor Activity of Ethanolic Extract from Thymbra Spicata L. aerial Parts: Effects on Cell Viability and Proliferation, Apoptosis Induction, STAT3, and NF-kB Signaling. Nutr. Cancer.

[24] Aleman R.S., Page R., Cedillos R., Montero-Fernández I., Fuentes J.A.M., Olson D.W., Aryana K. (2023). Influences of Yogurt with Functional Ingredients from Various Sources That Help Treat Leaky Gut on Intestinal Barrier Dysfunction in Caco-2 Cells. Pharmaceuticals.

[25] Pongkorpsakol P., Satianrapapong W., Wongkrasant P., Steinhagen P.R., Tuangkijkul N., Pathomthongtaweechai N., Muanprasat C. (2021). Establishment of Intestinal Epithelial Cell Monolayers and Their Use in Calcium Switch Assay for Assessment of Intestinal Tight Junction Assembly. Methods Mol. Biol..

[26] Khalil M., Piccapane F., Vacca M., Celano G., Mahdi L., Perniola V., Apa C.A., Annunziato A., Iacobellis I., Procino G. (2024). Nutritional and Physiological Properties of Thymbra spicata: In Vitro Study Using Fecal Fermentation and Intestinal Integrity Models. Nutrients.

[27] Woodland P., Batista-Lima F., Lee C., Preston S.L., Dettmar P., Sifrim D. (2015). Topical protection of human esophageal mucosal integrity. Am. J. Physiol. Gastrointest. Liver Physiol..

